# An observational study using eye tracking to assess resident and senior anesthetists’ situation awareness and visual perception in postpartum hemorrhage high fidelity simulation

**DOI:** 10.1371/journal.pone.0221515

**Published:** 2019-08-29

**Authors:** Arnaud Desvergez, Arnaud Winer, Jean-Bernard Gouyon, Médéric Descoins

**Affiliations:** 1 Centre d’Études Périnatales de l’Océan Indien (EA 7388), University Hospital of Reunion Island, Saint Pierre, La Réunion, France; 2 Centre de Simulation en Santé de l’Océan Indien, University Hospital of Reunion Island, Saint Pierre, La Réunion, France; 3 Critical Care Unit, University Hospital of Reunion Island Saint Pierre, La Réunion, France; 4 University of Reunion Island, La Réunion, France; University of Palermo, ITALY

## Abstract

**Background:**

The postpartum hemorrhage (PPH) is the leading cause of maternal mortality in the world. Human factors and especially situation awareness has primarily responsibility to explain suboptimal cares. Based on eye tracking and behavior analysis in high fidelity simulation of PPH management, the goal of this study is to identify perceptual and cognitive key parameters of the expertise.

**Methods:**

Two groups of fifteen anesthetists (residents and experienced anesthetists) watched the beginning of a severe simulated PPH management. During this first experimental phase, situation awareness was assessed using SAGAT (Situation Awareness Global Assessment Technique) questionnaire and visual behavior was analyzed with eye tracking. In the continuity of the video sequence, they have to step in the PPH situation and to provide care to the simulated patient. Performance of cares was evaluated and self-assessed as well as cognitive load.

**Results:**

No statistical difference between the residents and experienced anesthetists was observed on performance of simulated PPH management. The mean expected practice score was 76.9 ± 13.9%). Assessment of situation awareness (65 ± 7%), cognitive load (74.4 ± 11.3%) and theoretical knowledge of PPH (52.4 ± 3.5%) were also not statistically different between the two groups. Only results of self-assessed performance (respectively 66.1 ± 16.6 and 47.0 ± 20.8 for experts and residents) and eye-tracking data revealed that experts tended to get accurate evaluation of their performance and to monitor more the blood loss of the patient. Experts have in average 8.28% more fixating points than Novices and gazed the blood loss region longer (865 ms ± 439 vs. 717 ms ± 362).

**Conclusions:**

This study pointed out the limits of classical assessment of performance, and human factors based on questionnaires to identify expertise in simulated PPH care. A neuroscientific approach with new technology like eye tracking could provide new objective and more sensitive insights on human factors in simulated medical emergency situations.

## Introduction

The *postpartum* hemorrhage (PPH) is the leading cause of maternal mortality in the world [1–3]. In France, 20% of maternal deaths are attributable to PPH with a large regional disparity [4]. The French overseas regions (as the French Antilles, French Guiana or the Reunion Island) are particularly impacted with prevalence up to 10 times higher. In 2017, a survey estimated that 100% of maternal care was non-optimal with preventable deaths in 100% of cases [5]. Improving the management of this pathology remains a worldwide public health issue.

Retrospective real life case studies indicate a lack of recognition of the initial severity of patients and ineffective treatment choices, particularly concerning the anesthetic management of PPH (recognition and management of hemorrhagic shock, use of transfusion, anesthetic strategy) [6–8]. The analysis of the human factor in anesthesia resuscitation brings a new grid of reading within the framework of the complex and inter-professional management of this serious pathology.

The export of non-technical skills concepts from aeronautics to anesthesia has made possible to identify significant cognitive impairments during incidents in our practices [9]. Situational awareness (SA) is one of them [10,11]. It can be schematized by a three-level linear cognitive process starting from the integrated relevant information gathering in a dynamic environment (Level 1: Perception), an understanding of the situation (Level 2: Comprehension) and an anticipation of the clinical evolution of this situation which allows the elaboration of a management’s strategy (Level 3: Projection) and leads to the performance.

The dedicated analysis of this cognitive model in anesthesia accidents revealed its involvement in more than 80% of cases with a preponderance of the perception level [12,13]. A lack of information, particularly visual, leads to increase morbi-mortality of our practices. Our eye movements in dynamic situations are closer to cognition. Since the Yarbus’s study in 1956 [14], we know that it is a process controlled by the tasks in progress. The repetition of a correct oculomotor sequence is rewarded by dopaminergic circuits connected to visual attentional centers and is therefore selected by experience [15].

Expertise is a determinant of visual prospecting and performance management. We are convinced of the primary role of prospecting behavior and visual information gathering in the model of situation awareness and its involvement in the generation of incidents of anesthetic management [12,16]. We hypothesized that expert anesthetists develop specific visual behavior related to a higher level of situation awareness and performance than novices while maintaining superior cognitive reserve represented by a lower level of cognitive load.

The objective of this study is to characterize and compare the visual behavior of expert and novice anesthetists during a simulated severe PPH scenario and to assess levels of situation awareness, particularly perceptual, cognitive load and associated performance of simulated PPH management.

## Materials and methods

### Study design

This prospective observational study utilized a combination of quantitative and qualitative experimental research methodologies extracted from data recorded during experimental eye tracking and PPH sessions of high-fidelity simulation in healthcare. It was carried out from March to May 2016. This study was performed at the Healthcare Simulation Center of the Indian Ocean located in the University Hospital of La Réunion, France.

### Participants

30 anesthetists were divided into two equal groups. Novices group included residents with experience of less than 5 years and Experts group included anesthetists with more than 5 years of professional activity. All participants had normal or corrected to normal vision. After a general information session, the participants were required to review and sign a consent form that described the purpose and nature of the study as approved by The Ethic Committee of the CHU de La Reunion. Participants were free to participate and could withdraw at any time. No incentive was given.

### Conduct of the experiment

**Fig 1:**
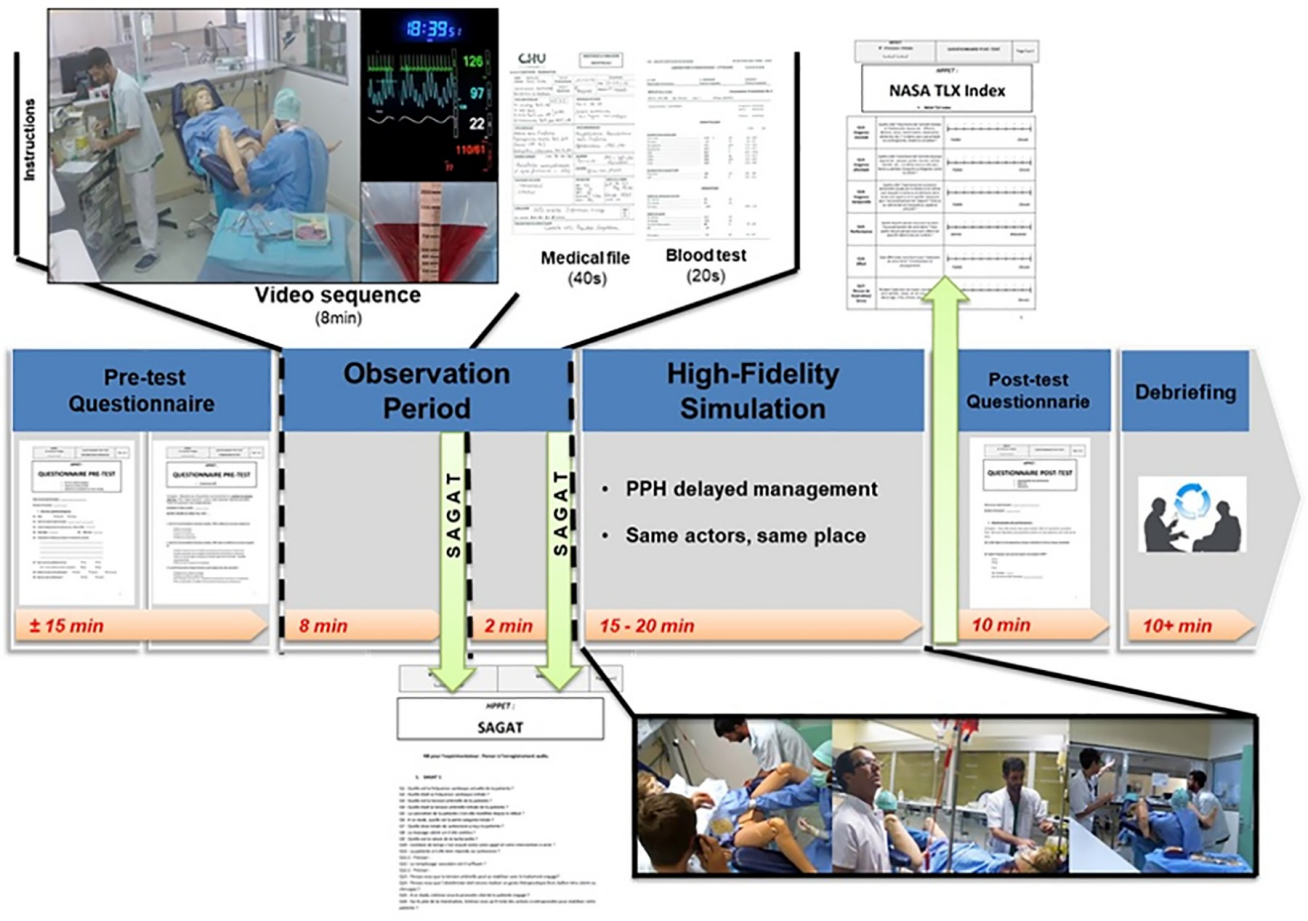
Description of the experimental protocol. First a pretest questionnaire is performed, it characterizes the physicians’ professional experience and the medical knowledge on PPH. After instruction slides, a video clip is presented to the anesthetist for oculometry. The story began at 6:30 p.m., he received a phone call from the nurse for a PPH in progress. The PPH situation presented on the screen (upper banner) showed Noelle^®^, the nurse and the obstetrician during the first 15 minutes of management without the anesthetist. The standardized environment is equipped as a conventional delivery room. The severity of PPH and the actors’ contemplative attitude associated with the inefficiency of the first treatments contributed to the clinical degradation of the patient. A SAGAT is performed at the end of the video. The questions concerned the hemodynamics profile, therapies already undertaken, and the complications envisaged for the patient. Then he had 40 seconds to consult the anesthesia file and 20 seconds to consult the blood test. Another SAGAT is then performed. The questions concern its HPP risk factors and its anesthetic management conditions (allergic risk, risk of difficult intubation, transfusion management). The anesthetist was then asked to manage the patient in the same simulated environment (lower band). Management must be intensive, but the situation was getting worse. The decision of hemostasis surgery marked the end of the scenario. A post-test self-assessment questionnaire and a NASA TLX Index conclude the session.

The experimental procedure is presented in Fig 1. The pretest questionnaires assessed the level of knowledge on PPH, the characterization of the anesthesia experience and previous exposure to high-fidelity simulation.

The eye-tracking procedure then proceeded for 8 minutes in a black room. They were to see the start of simulated PPH management that they will have to manage later. The rationale was to allow the participants to be fully aware of the PPH case they would have to manage during the high-fidelity simulation. The video featured a high-fidelity mannequin, a nurse and an obstetrician from delivery to the deterioration of the patient's clinical condition. Situation awareness was assessed by SAGAT questionnaire during this stage. The anesthetist started the high-fidelity simulation where the video sequence was interrupted matching with the call to the anesthetist. Cognitive coherence is guaranteed by the permanence of actors, time, places and material. The surgical decision ended the scenario. Final questionnaires provided anesthetist self-assessment and measurement of cognitive load by the NASA TLX index. An interview concluded experimentation.

### Eye tracking set up and analysis

**Fig 2:**
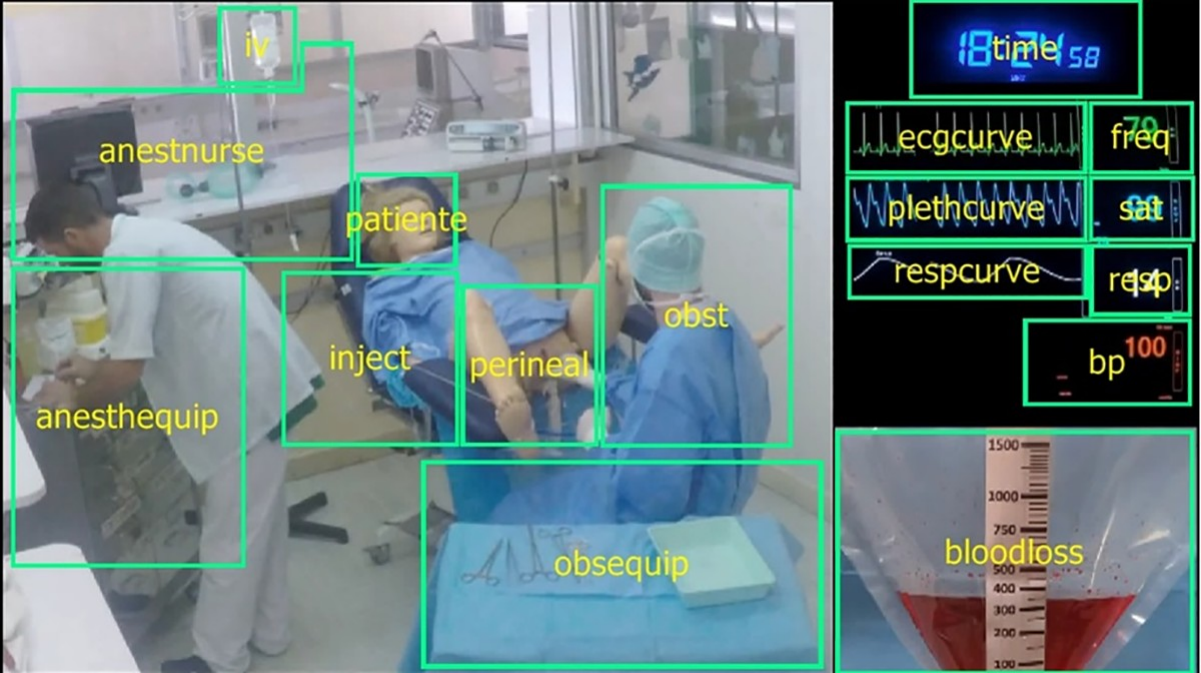
Screenshot presenting the video clip and the repartition of the region of interest. The two actors managed a simulated patient: the nurse on the left, the obstetrician on the right and the high-fidelity manikin in the center. Time, monitoring and quantification of bleeding were inserted on the right of the screen to improve the readability of the information and to better discriminate eye movements. The 17 yellow boxes correspond to the previously defined ROI. *“iv”*: corresponds to vascular filling fluids, *“anestnurse”*: nurse anesthesiologist, *“anesthequip”*: equipment of the anesthetist, *“patient”*: Noelle^®^ face, *“inject”*: injection site in the perfusion, *“perineal”*: perineal area, *“obst”*: obstetrician, *“obsequip”*: obstetrical material, *“time”*: hour of the action. *“Ecgcurve”*: heart rate curve, *“freq”*: heart rate, *“plethcurve”*: plethysmography curve, *“sat”*: oxygen saturation of the blood, *“respcurve”*: respiratory rate curve, *“resp”*: respiratory rate, *bBp”*: blood pressure, *“bloodloss”*: blood collection bag.

We used a fixed binocular eye-tracking device (The EyeTribe APS, Copenhagen, http://www.theeyetribe.com/) to measure the visuo-motor behavior of the participants and extract fixation points during viewing of a simulated PPH video. Participants' heads were placed 60 cm from a 22-inch LCD monitor with a resolution of 1920 by 1080 pixels (Philips 221B Brillance). An 8-minute video sequence was filmed in a fixed subjective view. It showed the 15 minutes before the anesthetists’ intervention thanks to the acceleration of non-determinant parts with six sequences related to the obstetrical gestures practiced and the patients clinical worsening (delivery, PPH diagnosis, call for an anesthetist, bleeding worsening, uterine revision and valve examination). As shown in Fig 2, the screenshot of the video provided clear access to the evolution of the time, patient’s bleeding and vital parameters.

With a sampling rate of 60 Hz, 2 points spaced 100 ms apart around a dispersion area of 50 pixels defined our fixation points. This experimental parameterization allowed good discrimination of objects larger than 1.20 cm, respecting the precision margins of the tracker [17]. Presentation, recording, data acquisition and definition of 17 regions of interest (ROI) were managed by the OGAMA v5.0.0 software (Open Gaze and Mouse Analyzer, Freie Universität, Berlin) [18]. Fig 2 shows the distributions of the 17 ROI. The variables extracted were the number of fixations (NBF) and the fixation duration mean (FDM) for each region of interest. Missing data were computed using MCMC method [19,20]. The attentional heat maps were extracted during key steps of care.

### Simulation of PPH

After watching the video clip, the anesthesiologists were asked to manage the simulated patient (high-fidelity mannequin Noëlle^®^ 57X.100, Gaumard) with the nurse and the obstetrician. The patient suffered from a hemorrhagic shock on severe PPH with delayed management. Their acting roles were normalized. The nurse followed the instructions of the anesthetist in the order of enunciation. The obstetrician made no therapeutic decisions. He had to continue the uterine massage and tell the anesthetist the persistence of the bleeding. No more help was available. Initially the care provided during the video part was composed of moderate fluid challenge and 30 UI oxytocin injection. Each resuscitation measure undertaken by the anesthetist induced a transient corrective response on the vital parameters of the patient. These responses were standardized and triggered by the simulation pilot. Whatever the actions provided, the trend continued to worsen. The surgery decision marked the end of the scenario. The scene was filmed for performance analysis.

### Data analysis

All data were registered with an anonymous inclusion number, and all questionnaires were filed by participants using a PhyMyAdmin database.

PPH performance was scored by a 16-item checklist (S1 Appendix) developed by a medical committee using professional recommendations. A percentage of bad practice was calculated when treatment was not administered at the recommended dosage or with incorrect prescribing rules.

Situation awareness was measured twice with the Situation Awareness Global Assessment Technique (SAGAT) questionnaire during the eye-tracking sequence (S2 Appendix). The 38-question SAGAT was achieved by an expert medical committee following the Goal Directed Task Analysis [21]. The numerical responses were accurate within a 10% range around the true value. We asked to justify the answers to the yes/no questions using keywords. The score was expressed as a percentage.

NASA TLX Index allowed the anesthesiologist to self-evaluate the level of cognitive workload after having been exposed to the situation [22]. Anesthetists’ self-evaluation of their knowledge, simulation performance, clinical understanding and their stress were done with Likert scales.

### Statistical analysis

Qualitative data are presented as counts and percentages whereas numerical ones as mean and standard deviation. To test the hypothesis that Experts are the most efficient and less sensitive to the cognitive workload than novices, simulation performance, NASA TLX Index and posttest self-assessments were subjected to parametric tests between the groups. Chi-square test was used for the qualitative variables and the student T-test for the quantitative. To test the hypothesis that the Experts have a higher SA level (particularly perceptual), a two-factor ANOVA (Group: Expert vs. Novices and SA Level: 1, 2 or 3) was made on SAGAT scores. In order to test the hypothesis that an “expert gaze” was applied to the video sequences of the PPH, the oculometric variables (respectively, number of fixation (NBF) and fixation duration mean (FDM)) were tested by ANOVAs on the two factors Groups and ROI (17 regions of interest). Post-hoc tests of Newman-Keuls have been applied to analyze significant effects. A result with P <0.05 was considered significant. All statistical analysis was computed using R v 3.3.1 software.

## Results

### Descriptive data

**Fig 3:**
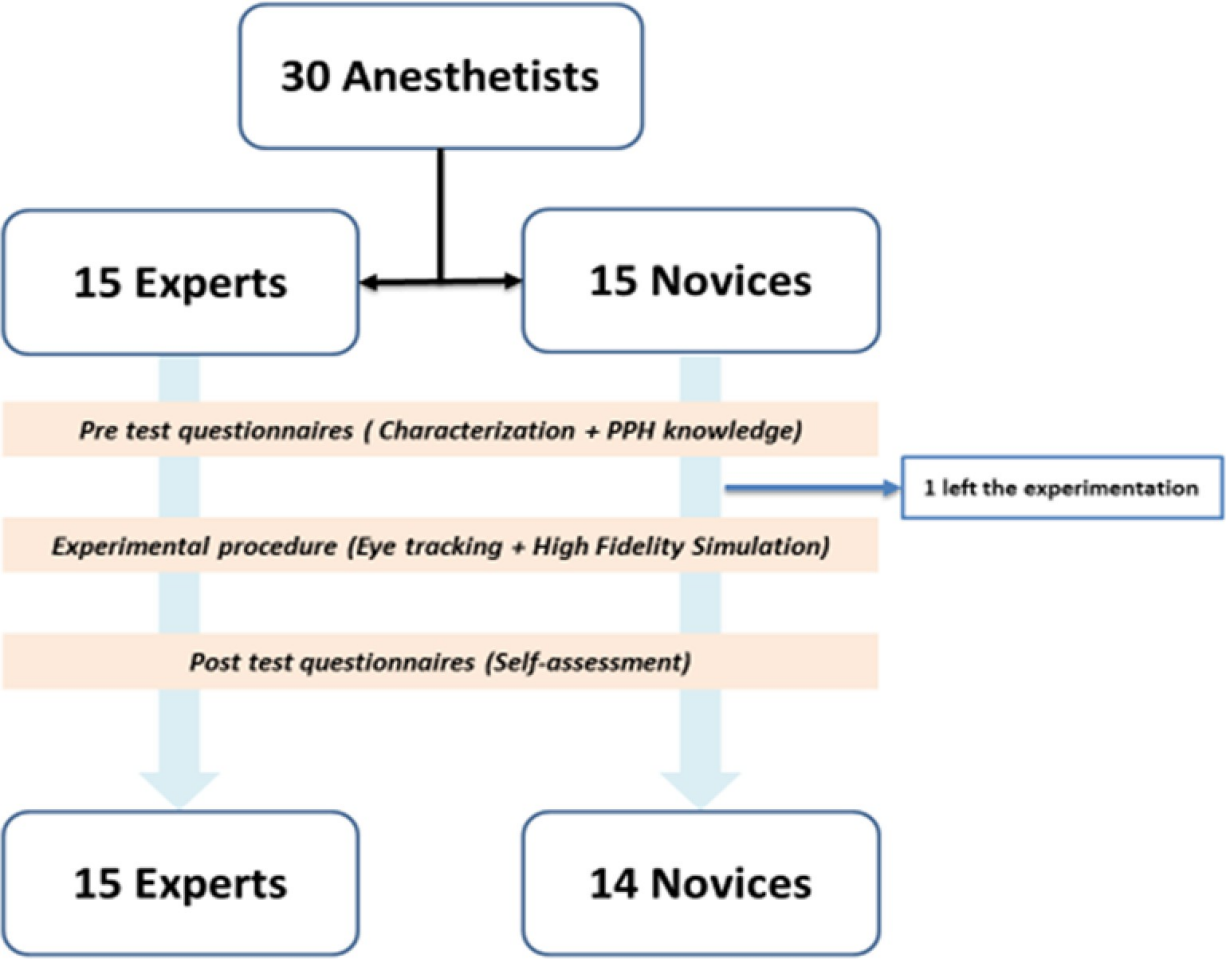
Experimental flow chart. 30 anesthetists participated in this experimental study. 15 experts with more than 5 years of experience. 15 novices with less than 5 years of experience. 1 novice left the experimental protocol before oculometry. The cumulative experience in maternity ward is significantly higher among Experts 12.1 ± 8.1 years versus 1.0 ± 1.7 years for Novices. Anesthetists’ level of theoretical knowledge on PPH is not statistically different between the groups (52.4% ± 3.5 of correct answers). The experts were confronted with more real PPH situation. They are 67% to have managed more than 50 cases and 27% more than 100 cases. Only 57% of the novices participated in the management of 10 to 50 cases for the most exposed (*p <0*.*05*). Participating anesthetists are familiar with high fidelity simulation since 67% of Experts (n = 10/15) and 87% of Novices (n = 13/15) have already practiced it. However, all the novices have already practiced PPH scenario while only 2 experts already did it (*p <0*.*001*). None of them practiced eye-tracking experiment before.

The 30 anesthetists included in this study were divided into two groups (Experts and Novices). Table 1 presents the demographic and professional characteristics of the participants. Fig 3 shows the study flow chart. One novice left the experimental protocol before ending.

**Table 1:** Characteristics of the participants.

	Total population (n = 30)	Experts (n = 15)	Novices (n = 15)	*p*
Hospital status: % (n)				***<0*.*001***^a^
- Hospital practitioner	46.6% (n = 14)	93.3% (n = 14)	0	
- Assistant	6.6% (n = 2)	6.7% (n = 1)	6.7% (n = 1)	
- Resident	46.6% (n = 14)	0	93.3% (n = 14)	
Percentage of the unit workforce:	69.7% (n = 30/43)	55.5% (n = 15/27)	93.7% (n = 15/16)	
Age (years μ ±σ)	34.5 ±8.5	41 ± 8	28 ± 2	***<0*.*001***
Gender % (n)				*0*.*709*
- Men	60% (n = 18)	53.3% (n = 8)	66.6% (n = 10)	
- Women	40% (n = 12)	46.7% (n = 7)	33.3% (n = 5)	
Vision trouble % (n) Vision corrected to normal % (n)	66.7% (n = 20) 66.7% (n = 20)	60% (n = 9) 60% (n = 9)	73% (n = 11) 73% (n = 11)	*0*.*449*
Experience in simulation % (n)				
- Low-fidelity	83.3% (n = 25)	66.7% (n = 10)	100% (n = 15)	***0*.*041***^b^
- High-fidelity	76.7% (n = 23)	66.7% (n = 10)	86.7% (n = 13)	*0*.*390*
- Including PPH	50% (n = 15)	6.7% (n = 2)	86.7% (n = 13)	***<0*.*001***^b^
Experience in Eye Tracking	0	0	0	
Cumulative experience: (years μ ±σ)				
- Anesthesia	8.1 ±8.9	14.9 ±8.0	1.3 ±1.0	***<0*.*001***^a^
- Maternity ward	6.5 ±8.1	12.1 ±8.2	1 ± 1.7	***<0*.*001***^a^
- Intensive Care unit	3.5 ± 4.6	6.1 ±5.2	0.8 ±0.8	***0*.*001***^a^
Resident: current assignment unit: % (n)				
- Anesthesia			53.3% (n = 8)	
- Medico-surgical ICU			40% (n = 6)	
- Neurosurgical ICU			6.6% (n = 1)	
Current experience in obstetrical anesthesia: % (n)				***0*.*002***^a^
- Very Often	13.3% (n = 4)	26.7% (n = 4)	0	
- Often	56.7% (n = 17)	73.3% (n = 11)	40% (n = 6)	
- Rarely	23.3% (n = 7)	0	46.7% (n = 7)	
- None	6.7% (n = 2)	0	13.3% (n = 2)	
Real cases of PPH managed: % (n)				***<0*.*001***^a^
- > 100 cases	13.3% (n = 4)	26.7% (n = 4)	0	
- 50 to 100 cases	20% (n = 6)	40% (n = 6)	0	
- 10 to 50 cases	43.3% (n = 13)	26.7% (n = 4)	60% (n = 9)	
- < 10 cases	23.3% (n = 7)	6.6% (n = 1)	40% (n = 6)	
Night shifts per month (μ ± σ)	4.17 ±1.11	3.40 ±0.63	4.93 ±0.96	***<0*.*001***
Patients managed per week (μ ± σ)	23.8 ±13.2	26.8 ±6.6	20.8 ±17.2	*0*.*223*
Worked hours per week (μ ± σ)	49.7 ±5.6	46.9 ±3.2	52.6 ±6.0	***0*.*004***

The table shows the characteristics of the population. Experts are senior doctors who have completed their studies. Novices are doctors in specialization in anesthesia and intensive medicine.

^a^The real and felt experience in obstetric anesthesia is higher among senior physicians. All doctors have already practiced simulation.

^b^Residents experienced hemorrhage scenarios more frequently than seniors because of their training.

### PPH management performance

The Experts did not show a significantly better performance than the Novices, nor in terms of the percentage of expected therapeutic actions (80 ± 8.90% with 10.86 ± 6.25% of bad practices), nor in terms of simulated care duration (19:09 ± 04:45 min). Whereas novices did 73 ± 17.54% of expected therapeutic actions including 16.13 ± 13.34% of bad practices in 18:15 ± 02:44 minutes.

### Oculometric data

**Fig 4:**
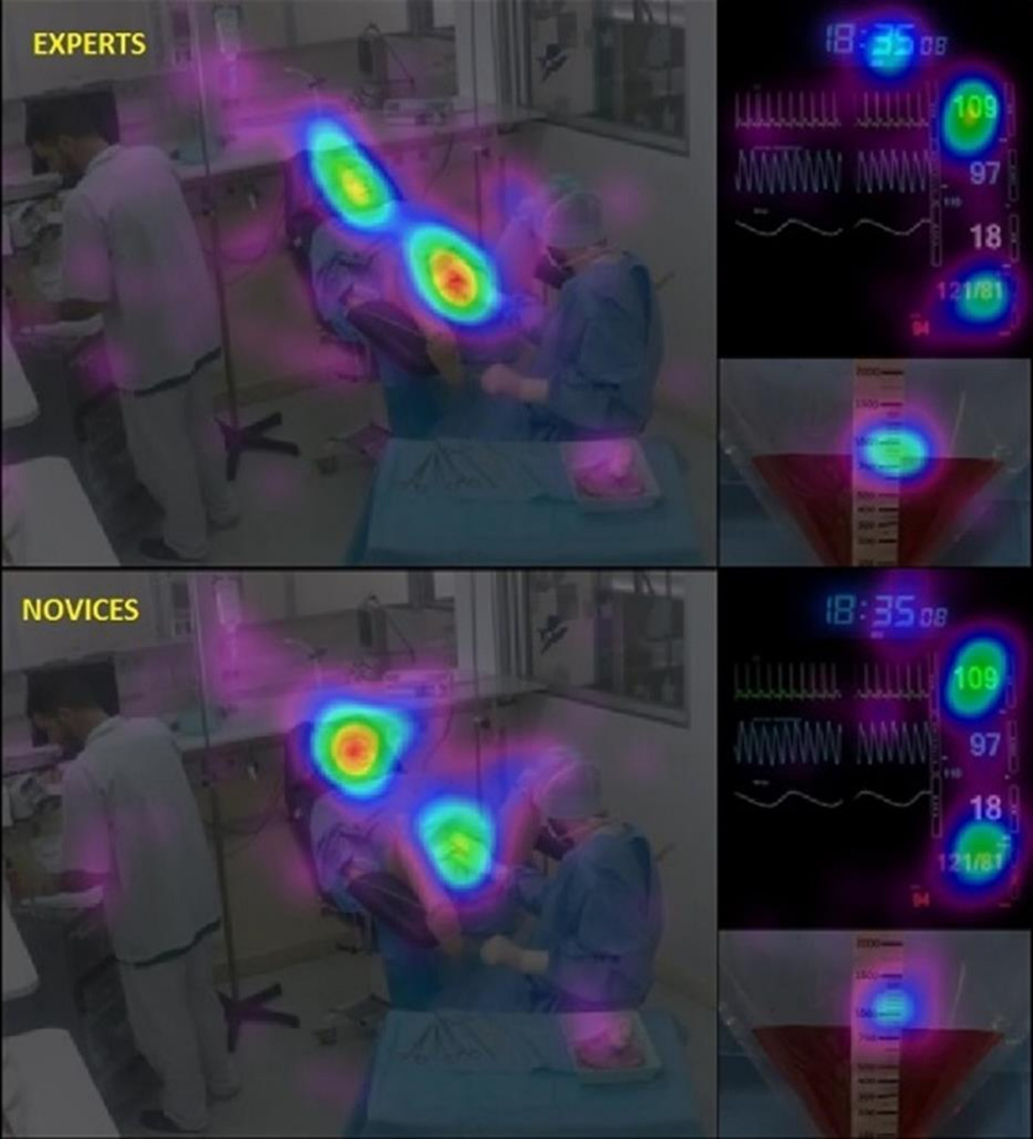
Heat map during the uterine revision step. Heat map calculated from anesthetists’ fixation points on video during the uterine revision step. The hotter the point (from blue to red), the greater the cumulative fixation time in the region of interest. Unlike the Novices (bottom), the Experts (above) showed a greater distribution of the gaze on the different elements of the scope. The interest in time and the blood collection bag appeared more important among the Experts. They paid a lot of attention to the perineal area whereas the Novices looked at Noelle’s face instead.

**Fig 5:**
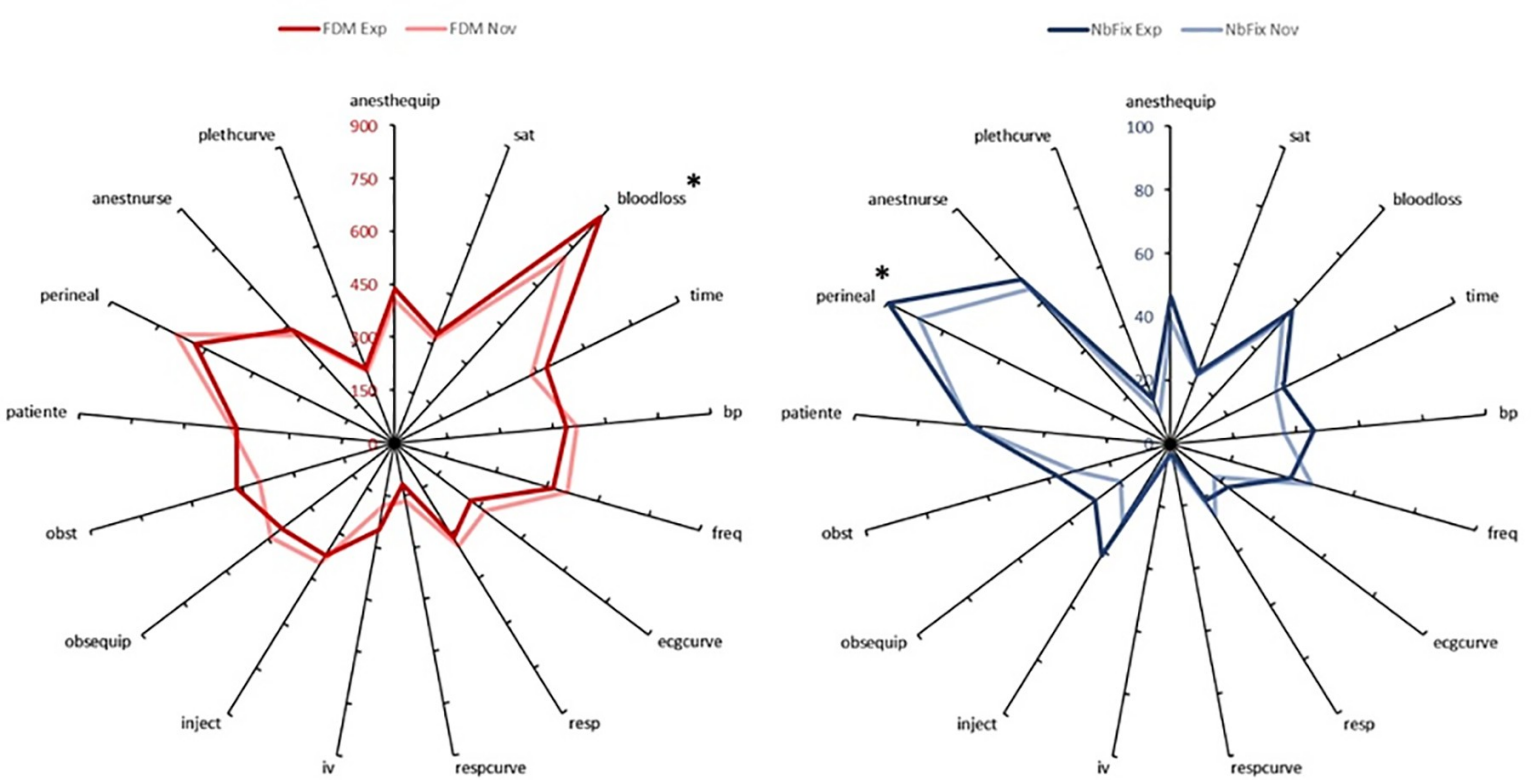
Graphical representation of the fixation number and fixation duration means of Experts and Novices according to the ROI. Presentation of the eye-tracking data by ROI according to the group (Experts or Novices). The graph on the left shows the fixation duration mean (FDM). Experts gazed on average more the ROI “bloodloss” 865 ms compared to 717 ms in Novices. The graph on the right shows the number of fixation (NBF). The post-hoc analysis revealed only difference between Novices and Experts on the ROI “perineal”. The experts will more often gaze this region than the novices (99 versus 89). The overlap of these curves makes it appear that the ROI most often viewed are not those which are viewed the longest.

Overall recording, the missing data rate is 5.17%. 2958 fixation points were computed for analysis. The observation of attentional heat map showed different gaze dispersion between the experts and the novices. Fig 4 shows an example of heat map extracted during the uterine revision phase. Experts paid more attention to monitoring information. As shown in Fig 5, Experts look more often at the ROI “perineal” and look at the ROI “bloodloss” longer than novices (*p <0*.*05*). In addition, the most frequently watched ROIs are not those gazed the longest on average.

### Number of fixation analysis

Analysis of the eye-tracking data revealed that Experts have in average 8.28% more fixating points than Novices (in average per participants respectively n = 791.4 vs. 725.9, F (1, 2754) = 14.5; *p <0*.*001*). Specific elements of the environment (effect of the factor ROI) are more often fixated, revealing a higher interest for anesthetists (F (16, 2754) = 155.4; *p <0*.*001*). The 5 most frequently watched ROIs were the perineal region (15.8 ± 0.6), the nurse (10.8 ± 0.5), the patient (10.6 ± 0.4), the blood loss (9.3 ± 0.4) and heart rate (7.2 ± 0.3). The post-hoc analysis of the significant interaction effect between the factors Group and ROI (F (16, 2754) = 2.22; *p = 0*.*003*) revealed that experts showed a superior interest for the perineal region. The post-hoc analysis of the interaction effect between the Group and ROI factors (F (16, 2754) = 1.82, *p <0*.*05*) only revealed that Experts fixed the perineal region significantly more often than the Novices (16.7 ± 0.44 vs. 14.9 ± 0.45, *p <0*.*05*), no other ROI were statistically more observed by Experts compared to Novices.

### Mean duration of the fixation

Anesthetists had an average time of 414 ± 182 ms (ranging in average from 780 to 153ms) by areas of interest and there is no main effect of the factor Groups was observed (F (1, 2744) = 0.43 p = ns). All the ROI were not fixed with the same interest (F (16.2601) = 70.7 *p <0*.*0001*). Post-hoc analysis showed that the 5 ROIs with the highest average fixation duration are blood loss (790 ± 410 ms), significantly observed longer than the perineal region (663 ± 222 ms). Without any difference between Blood pressure (506 ± 258 ms), heart rate (494 ± 260 ms) and the patient (461 ± 215 ms), these regions are significantly gazed for a longer time. The interaction between the factors Group and ROI (F (16.2601) = 2.18, *p <0*.*05*) and post-hoc analysis only revealed that Experts did not monitor blood loss like novices. Experts gazed significantly longer blood loss ROI than Novices (865 ms ± 439 vs. 717 ms ± 362, p <0.001).

### Situation awareness assessment

The global level of situation awareness has been evaluated with the SAGAT score at 64.8 ± 6.4% among anesthetists. Surprisingly, Experts did not statistically reach a significant higher level of situation awareness (66.3 ± 7.2% vs. 63.1 ± 5.1% respectively, p = ns). The level of situation awareness is burdened by the item comprehension (SA Level 2 = 51.1 ± 8.3%) which is significantly lower than perception and projection levels (SA Level 1 = 70.6 ± 10.6% and level 3 = 72.3 ± 9.7%) (F (2.81) = 43.32, *p <0*.*001*).

### Post-test anesthetist self-assessment

#### Workload and realism of the situation

Anesthetists found the simulated situation realistic (80.1 ± 18.4%) and immersive (79.5 ± 15.1%). Because of the lack of help and the severe condition of the patient, the simulated scenario has a very high level of cognitive load. In this experimental simulated PPH scenario, Experts and Novices have experienced a high level of cognitive load with no significant difference between groups (74.3 ± 11.4% vs. 76.6 ± 11.7%, respectively).

#### Feeling of personal competence

As shown in Table 2, post-test self-assessment revealed a significant discrepancy between objective evaluation and subjective feelings in groups. Experts reported a greater feeling of performance and knowledge than Novices (self-assessment of performance 66.1 ± 16.6 vs. 47.0 ± 20.8, *p <0*.*05*, self-assessment of medical knowledge 73.8 ± 9 vs. 52.1 ± 19.9, *p <0*.*05*).

**Table 2:** PPH knowledge, PPH simulation performance and anesthetist post-test self-assessment.

	Total population (n = 30)	Experts (n = 15)	Novices (n = 14)	*p*
PPH knowledge score (%)	52.4 ±3.5	52.1 ±3.4	52.7 ±3.6	*0*.*645*
Self-assessment of knowledge level (%)	63.3 ±18.8	73.8 ±9.0	52.1 ±19.9	***0*.*002***
SAGAT Total score (%)	66.5 ±7.1	68.1 ±8.6	64.8 ±4.7	*0*.*200*
Self-assessment of the effectiveness of information taking	60.7 ±16.5	66.7 ±11.2	54.3 ±19.1	***0*.*046***
Self-assessment of understanding of the clinical situation	71.5 ±18.4	80.0 ±7.3	62.4 ±22.3	***0*.*013***
Simulation performance:				* *
- Expected practices (%)	76.9 ±13.9	80.0 ±8.9	73.7 ±17.5	*0*.*22*
- Including bad practices (%)	13.4 ±10.5	10.9 ±6.3	11.6 ±5.7	*0*.*73*
Simulated care duration (sec)	1123 ± 232	1149 ± 285	1095 ± 164	*0*.*53*
Self-assessment of overall management performance	56, 9 ± 20.8	66.1 ±16.6	47.0 ±20.8	***0*.*012***
Self-assessment of the performance of the resuscitation	60.8 ±21.1	70.0 ±14.4	51.0 ±23.1	***0*.*015***
Self-assessment of commitment in care	88.6 ±13.4	91.0 ±7.2	86.1 ±17.8	*0*.*348*
NASA TLX Index score (%)	74.4 ±11.3	74.3 ±11.4	74.6 ±11.7	*0*.*947*
Self-assessment of perceived stress level in simulation	62.5 ±19.8	59.2 ±21.1	66.0 ±18.4	*0*.*362*
Self-assessment of general fatigue level	60.4 ±23.3	62.2 ±28.1	58.5 ±17.6	*0*.*673*
Self-assessment of realism level	80.1 ±18.4	80.8 ±21.3	79.3 ±15.6	*0*.*828*
Self-assessment of immersion level	79.5 ±15.1	85.2 ±10.3	73.4 ±13.4	***0*.*039***
Self-assessment of motivation level to participate in the study	81.9 ±17.7	90.1 ±8.5	73.1 ±20.9	***0*.*011***
Self-assessment of satisfaction level	86.0 ±21.0	87.1 ±22.5	84.7 ±20.1	*0*.*762*

Comparison of theoretical knowledge, management performance, level of situational awareness, cognitive load measured and felt. There is a tendency for seniors to overestimate their performance compared to residents when there is no observable difference measured. The clinical situation was considered very realistic by the doctors. Seniors rated their level of immersion and motivation superior to residents.

## Discussion

This is the first study linking a visual behavior analysis using a standardized fixed eye tracking protocol, performance and human factors in high-fidelity simulation of PPH. Our main result is the identification of an expert visual behavior characterized by an increased number of total fixation points in comparison to residents. The study also shows the interest of eye tracking in assessing Situation Awareness at the perceptive level.

The eye-tracking protocol allowed us to analyze the visual perception objectively and to add a cognitive dimension since eye movement is a cognition-dependent process [15]. Experts have developed 3.5% more fixation points than novices. This increased number of fixations seems to be a marker of expertise in dynamic situations and appears to correspond to a more frequent updating of critical data related to the clinical need. This observation is also found in aeronautic with a 10% increase in the fixation points for expert pilots on landings [23]. However, it has been reported in medical radiology that during a chest X-ray analysis, the expertise is characterized by a reduction in the number and times of fixation [24]. But in this case—a static diagnostic task—the cognitive demand is not the same as for a changing dynamic environment requiring continuous reassessment.

Fixation time is a neurophysiological driven variable. Difference as the additional 150 ms (30% of the average fixation mean) spent by experts compared to novices for evaluating the bleeding loss mean clearly different perception and cognition processes. This is a major observation because the lack of recognition and anticipation of the bleeding problem contributes to degrading the level of maternal care and creates morbi-mortality [6,8]. Evaluation of bleeding is the center of the cognitive strategy put in place by experts to monitor the dynamic of the PPH. It results in a modification of the visual behavior and a specific cognitive treatment at different levels of integration (perception, comprehension, projection and probably the performances).

Their apparent interest for the perineal region raises questions. We make several assumptions. First, it can be a center bias, described in experimental human and animal studies [25]. This may reflect a physiological bias to return the eyes to the center of the orbits and in our eye tracking protocol to the center of the screen where the perineal region is located. Second, current research tends to show that experts use their peripheral vision more. Experienced endoscopists showed greater ability to detect adenomas when they looked at the central part of the screen, it is thus possible to extract information by maintaining a central position of the gaze and triggers a saccade when an unexpected event appears [26]. On sports fields, researchers proved that expert basket players retained a better awareness of the game than novices with a hidden foveal vision attesting that the peripheral vision of the expert can support complex integrated cognitive processes [27]. In addition, experts could have a more efficient information extraction capacity; this cognitive economy would leave more time to control gynecological procedures and can explain this center fixation tendency to the perineal region. Further studies would be needed to investigate the attractiveness of the different ROI. For example, using a different location of the ROI in the video sequences or a different point of view could add a clear ranking of the ROI in PPH management.

In a clinical simulated dynamic situation, it is interesting to see what are the cognitive determinants of clinical sense during the assessment of the hemorrhagic shock. Indeed, the analysis of visual behavior reveals processes of inhibition of some distractors (Perineal, Nurse, Patient) which are often fixed but not long observed for lack of useful information. While the anesthetists spend more time in extracting information on clinically relevant regions of their environment (Blood Loss, Heart Rate, Blood Pressure).

At first reading, the present study of human factors was disappointing. No difference was observed between anesthetist students and experienced professionals while the SAGAT, we have developed, was an arduous 38-question SAGAT (including 19 perceptive, 9 understanding, 10 anticipatory questions with justifications requested for yes/no questions) which was supposed to be more discriminant. The overall SAGAT score was 65 ± 6%. Compared to Stratton’s study on residents with sleep deprivation it was lower [28]. Indeed, in a traumatic resuscitation and respiratory distress simulated exercise, with a 10-question SAGAT administered at 3 minutes of simulation, they describe an average situation awareness value of 80%. In other nursing studies in simulated emergency settings, low SA scores were identified, averaging around 53% across scenarios and group [29]. The apparent weakness of comprehension (SA level 2 = 51%) in our SAGAT analysis is surprising where we expected a lack of perception (SA level 1 = 71%) according to the literature (11,12). A nursing study on a hypovolemic scenario also found a low level of comprehension at 29% with 2 yes / no questions asked at random times [30]. Other experimental studies tend to find perceptual failures. This weakness probably comes from the way the SAGAT questionnaire was administered. We have favored a low level of intrusion during the 8-minute video at the expense of a probable memory bias [31].

In addition, the expertise should have protected the experts from elevated cognitive load [32,33] and should have allowed better situation awareness [11,34]. Once again, the tools described in the literature (SAGAT and NASA TLX Index) were not able to discriminate our groups whereas self-assessment questionnaires allowed it. In the simulation scenario, we suppose that, for both groups, the delay of care generated a cognitive overload limiting the discriminating capacity of the NASA TLX. Current findings suggest that physiological indices are the most sensitive means for detecting variations in cognitive load while self-reporting assessment have poor validity score (1.40 ± 0.58 in medical education vs. 1.71 ± 0.70 for physiological measurements) but may not be sufficient to guarantee a better clinical patient’s outcome and physician’s performance in the real clinical practice [35]. Complementary eye tracking data analysis as measurement of the pupillary diameter could have added to our questionnaire assessment of cognitive load a physiological dimension. Indeed changes in pupillary diameter reflects adrenergic activity and cognitive load [36]. Other methods such as real-time EEG show promising results with very strong discrimination power [37,38].

This study clearly showed the effect of expertise in PPH management at the perceptual level with the eye-tracking data. However, there is currently a critical lack of efficient tools to provide insight of the behavioral level and of the cognitive mechanism at work (situation awareness and cognitive load). Understanding this discrepancy is a major issue.

Evidence of the interest of simulation-based training in improving physicians’ performances are clear both for initial training and for maintaining skills [39–42]. A meta-analysis found an improvement in professional practices in the field of obstetric emergencies [43]. Simulation based training has become a national recommendation in France. Our pre-graduate residents are trained since the first year to manage obstetric incidents through simulation. Moreover, since 2013, all the centers in our region have adopted a unique protocol gathering the PPH management’s key point in the form of a checklist with cognitive aid (see S3 Appendix). It’s probably for these reasons that we did not show any statistical difference in performance between Experts and Novices despite the higher variability of the Novices performance; a marker of learning processes [44]. For example, during our experimental phase, we observed the case of a novice (second year resident) with the same level of theoretical knowledge as the participants who had never participated in a real PPH management. He was just exposed to 1 PPH scenario in high fidelity simulation the month before. In our protocol, these novices performed the same level of performance as practitioners with clinical experience of more than 100 PPH cases. Can we imagine this undergraduate resident alone on commands for a maternity night shift? Even though the evidence of the ecological validity of the simulation is growing [45,46], the issue of performance extrapolation still remains. Based on the clinical expertise we do not think that novices are competent enough but on the move to expertize. Our assessment of performance with checklist is not sufficiently discriminating. A review published in 2001 reported that the efficacy of methodologies for assessment of performance during the simulation was largely undetermined [47]. Behavioral and NTS analysis suffers from the same limitations. It would even seem that adding NTS training appears not to substantively enhance simulation-based learning [39]. Observational scales are used and there is still a lack of research examining performance transfers, sustainability, and direct patient outcomes [48]. Even though we know that they contribute to poor performance, we cannot conclude that NTS simulation training improves the safety of the patient.

In the present study, self-assessment of performances could be closer to a good assessment of the care capacity of our participants. It was much lower among novices (sometimes up to 20% lower). We assume that this difference comes from a higher self-confidence associated with better self-efficacy of experts. Indeed, considering the determinants of self-efficacy [49]: clinical successes, vicarious experience, social persuasion and physiological states, experts tended to self-assess more accurately. Eventually, the accuracy of self-assessment would be a good target marker of the expertise.

There is a need to update simulation assessment with behavioral tools. It is difficult to think that a complex performance score based on behavioral data would be less efficient than self-assessments. We should be able to create such a score soon.

Our study has some limitations. The number of anesthesiologists included in each experimental group may seem low in the context of simulation study. No a priori calculation of the sample size was sufficiently reliable due to the mixed experimental design using a first PPH video watching session and the second one involving the participants in the management of the watched PPH. However, in regard of the Tien et al. [50] review of 7360 studies using eye tracking for skills assessment and training, we included more participants than the average 22.3+/-6.3 reported from the literature. Second, our anesthesiologists originated all from the same center and the regional PPH management protocol and training program could have a homogenization effect over practitioners. The additions of these two limitations would have resulted in the non-significant statistical analysis on PPH management score and SAGAT. Despite large size effects calculated for each variable consolidating our interpretations, complementary multicenter study included more participants are needed.

## Conclusion

Simulation assessment tools as performance score, SAGAT and NASA TLX have shown their limits in this study in terms of sensibility and discriminating power. This study questioned the development of healthcare high-fidelity simulation as a certification tool for medical skills and competencies. The contributions of new technologies (as portable Eye Tracking and EEG) to better characterization and recognition of expertise will have to be addressed.

Beyond the special case of PPH management, this study suggests the need for an innovative neuroscientific approach to better understand the cognitive processes that support medical expertise in lifesaving emergencies.

## Supporting information

S1 Appendix Checklist for PPH management performance. Left column: the actions expected for the PPH management in this case. Right column: the action has been done but with a technical error (drug, dosage or procedure).
(PDF)

S2 Appendix Situation Awareness Global Assessment Technique (SAGAT) used in this protocol. This 38-questions SAGAT (including 19 perceptive, 9 understanding, 10 anticipatory questions with justifications requested for yes/no questions) was designed to be hard and discriminant. It was administered during the video sequence just before anesthetist’s intervention.
(PDF)

S3 Appendix Regional protocol for PPH management. This algorithm is a checklist and serves as support for PPH management in Reunion Island. It helps to remember the steps of management and each expected action is checked and dated when performed.
(PDF)

S1 File Eye-tracking dataset. (XLSX)

S2 File SAGAT questionnaire dataset. (XLSX)

S3 File General characteristics of the participants dataset. (XLSX)

S4 File Self-assessment questionnaires dataset. (XLSX)
