# Supplementary material for: An observational study using eye tracking to assess resident and senior anesthetists’ situation awareness and visual perception in postpartum hemorrhage high fidelity simulation
Source: PLoS One. 2019 Aug 29;14(8):e0221515. doi: 10.1371/journal.pone.0221515 (PMC6715225; doi:10.1371/journal.pone.0221515)
Supplement: S1 Appendix — Left column: the actions expected for the PPH management in this case. Right column: the action has been done but with a technical error (drug, dosage or procedure). (PDF) [file pone.0221515.s001.pdf]

**EXPECTED THERAPEUTICS ACTIONS**		***BAD PRACTICES***
**Resuscitation**		
Second IV line	□	□
Blood Test: blood count, hemostasis, fibrinogen	□	□
Fluid challenge: 500 ml crystalloid + 500ml colloid	□	□
Vasopressors: Ephedrine or Neosynephrine, any dosage, IV	□	□
Trendelenburg position	□	□
Oxygen therapy: any dosage	□	□
Total: _/6		Total: _/6
**Prevention of coagulopathy**		
Antifibrinolytic: Tranexamic acid 1g on 20min	□	□
Fibrinogen: 3g IV	□	□
Calcium gluconate: 2g IV	□	□
Warm up: any way	□	□
Total: _/4		Total: _/4
**Transfusion**		
Order blood product: red blood cell and frozen plasma	□	□
Transfuse	□	□
Total: _/2		Total: _/2
**Uterotonic**		
Sulprostone: 500µg in 50ml on 1h	□	□
Total: _/1		Total: _/1
**Antibiotics**		
Dalacine: 600mg IV	□	□
Total: _/1		Total: _/1
**Generals measures**		
Call for PPH trolley	□	□
Call for help	□	□
Total: _/2		Total: _/2

TOTAL of EXPECTED ACTIONS: _/16	*Including bad practices: _/16*
