# Supplementary material for: An observational study using eye tracking to assess resident and senior anesthetists’ situation awareness and visual perception in postpartum hemorrhage high fidelity simulation
Source: PLoS One. 2019 Aug 29;14(8):e0221515. doi: 10.1371/journal.pone.0221515 (PMC6715225; doi:10.1371/journal.pone.0221515)
Supplement: S2 Appendix — This 38-questions SAGAT (including 19 perceptive, 9 understanding, 10 anticipatory questions with justifications requested for yes/no questions) was designed to be hard and discriminant. It was administered during the video sequence just before anesthetist’s intervention. (PDF) [file pone.0221515.s002.pdf]

SAGAT

1. SAGAT 1 (After watching the video sequence)

Q1: What is the patient’s current heart rate?

Q2: What was the patient’s initial heart rate?

Q3: What is the patient's blood pressure?

Q4: What was the patient’s initial blood pressure?

Q5: Has the patient's saturation changed since the beginning?

Q6: At this stage, what is the total blood loss?

Q7: What total dose of Oxytocin did the patient receive?

Q8: Has the uterine massage been continuous?

Q9: What is the reason for tachycardia?

Q10: How much time has passed between your call and your upcoming intervention?

Q11: Did the patient respond well to Oxytocin?

Q11.1: Specify:

Q12: Is the vascular filling enough?

Q12.1: Specify:

Q13: Do you think that blood pressure can stabilize with the treatment started?

Q14: Do you think that the obstetrician must still perform a therapeutic procedure (excluding intrauterine balloon or surgery)?

Q15: At this stage, do you consider the vital prognosis of the patient involved?

Q16: In terms of resuscitation, do you think that there are still actions to be undertaken to stabilize your patient?

2. SAGAT 2 (After consultation of the anesthesia file and the blood test)

Q17: What is the patient’s gesture and parity?

Q18: How much vaginal delivery did she get?

Q19: Does the patient have allergies?

Q19.1: Specify:

Q20: Is the current pregnancy normal?

Q20.1: Specify:

Q21: Does the clinical examination have any special features?

Q21.1: Specify:

Q22: What is the patient’s blood type?

Q23: On the biological assessment, what is the patient’s hemoglobinemia?

Q24: On the biological assessment, what is the patient’s platelet count?

Q25: What are the risk factors for PPH presented by the patient?

Q26: What information does the analysis of patient’s hemostasis bring you?

Q27: Why a blood test was prescribed at the consultation?

Q28: Which blood product or blood derivative do you think you need to transfuse as soon as possible?

Q28.1: Why?

Q29: Do you consider it necessary to contact the blood deposit quickly?

Q29.1: Why?

Q30: In case of clinical deterioration, if you decided to intubate this patient, would you consider this intubation difficult?

Q30.1: Why?
