# Supplementary material for: An observational study using eye tracking to assess resident and senior anesthetists’ situation awareness and visual perception in postpartum hemorrhage high fidelity simulation
Source: PLoS One. 2019 Aug 29;14(8):e0221515. doi: 10.1371/journal.pone.0221515 (PMC6715225; doi:10.1371/journal.pone.0221515)
Supplement: S3 Appendix — This algorithm is a checklist and serves as support for PPH management in Reunion Island. It helps to remember the steps of management and each expected action is checked and dated when performed. (PDF) [file pone.0221515.s003.pdf]

PRISE EN CHARGE REGIONALE DE L'HPP

Repère — Réseau Périnatal Réunion

Heure du diagnostic

Modalité d'accouchement
Voie basse / Césarienne
Volume pertes sanguines :

	FAIT	HEURE
Scope		
Monitorage PA		
SpO2		
1ère Hemocue		
Appel CTS		

(mise en réserve culots)

Appel immédiat
Obstréticien de garde : 5.4396
Anesthésiste : 5.4397
Interne gynéco : 5.4870
IADE : 5.4167

	FAIT	HEURE
Sac de recueil		
DA+RU		
ATB(1)		
Exam sous valves		

	FAIT	HEURE
Vidange vésicale		
Massage		
Expression		
SYNTO®(2)		

Si PABAL : NALADOR d'emblée

	FAIT	HEURE
O2(6l)		
Trendelenburg		
Réchauffer		
Remplir		

(Ringer/serum salé iso)

	FAIT	HEURE
2ème VVP + Valve anti retour		
1er bilan urgent(3)		
EXACYL®(4)		
GLUCONATE DE CA(5)		

30 MINUTES → Traitement efficace → Surveillance : 2 heures supplémentaires
Expression utérine / vérifier tonicité

Volume
Pertes sanguines

	FAIT	HEURE
Sonde U à demeure		
Diurèse horaire		
Trendelenburg		
O2(6l)		
Hémocue : 1/H		
Prévenir bloc		

(si voie basse)

	FAIT	HEURE
Arrêt SYNTO® ▼		
NALADOR®(6)		

Sur valve anti retour

Si PABAL : NALADOR d'emblée

	FAIT	HEURE
2ème bilan urgent (7)		
CLOTTAFACT®(8)		
Culots globulaires		

	FAIT	HEURE
Maintien PAM		
Remplissage (Plasmion/Voluven)		
Alerte RX vasculaire Ou Alerte CHU :		

Ballonnet BAKRI	Si pose : Heure :

60 MINUTES → Traitement efficace → Surveillance : 2 heures supplémentaires
Expression utérine / vérifier tonicité

Volume
pertes sanguines

Heure de départ au bloc : H

Chirurgie conservatrice
- Plicature, capitonnage ☐
- Ligatures vasculaires ☐

Remplissage ☐
Néosinéphrine, Ephédrine, Noradrénaline ☐
Poursuite culots ☐
PFC ☐
Concentrés plaquettaires ☐
NOVOSEVEN(9) ☐

Hystérectomie d'hémostase

Si embolisation	Si transfert
Remarques :	Accord SAMU ET centre receveur
**LIAISON GO et MAR**	
Heure départ Rx interventionnelle :	Heure décision :<br>Heure départ :<br>Centre receveur :

Si transfert : copie du recto et verso à remettre au centre receveur

---

(1) Antibiothérapie
Augmentin® : 2g perfusette
Dalacine® : 600.mg dans 50ml en 20mn si allergie

(2) SYNTOCINON® — Frigo
5 à 10 Ui en IVD puis 5 à 10 UI en IVL pendant 2 heures

(3) 1er BILAN :
Vérifier groupe/RAI
(NFS, plaq, TP, TCA, Fibrinogène, PDF, + 1 tube sec pour Test Coagulopathie, iono, calcémie ionisée)

(4) EXACYL®
15 mg/Kg
1g en IVL sur 20mn puis 1g/8H puis STOP

(5) GLUCONATE DE CA
2 AMP en IVL sur 4 à 5 mn (robinet distal)

(6) Sulprostone NALADOR®
1ère amp de 500µg dans 50ml : PSE 50ml/H
2ème amp de 500µg dans 50ml : PSE 10ml/h
3ème amp en fonction de l'évolution

(7) 2ème BILAN
idem 1er bilan + Troponine

(8) CLOTTAFAC® — Frigo
2 à 3 flacons de 1,5g

Seuils à atteindre
Fibrinogène 1-3gr (pour Fg>1.5-2g/L)
CG : pour Hb 9-10g/dL
PFC : ratio= 1 pour1 CG
Plaquettes dès 5 CG (pour >75-100 G/L)
CaCl2 1 ampoule IVL (pour 5 CG)

(9) NOVOSEVEN®
60 à 90µg/kg (que si hémostase biologique contrôlée)

Numéros utiles :
Radio embolisateur : journée : 5.5858 / Nuit et jour férié : appel du radiologue par standard
SAMU : 15 puis 3 puis 1 (cf. protocole appel SAMU)
CTS : Tél. 54130 Fax : 0262 35 58 19

GYNECO CHU NORD : 0262 90 55 34 (salle d'acct)
Demander le BIP du gynéco de garde
MAR Salle d'urgence : 0692 26 76 57

Version du 15/04/2015
